# Reasons for the Formation and Properties of Soliton-Like Charge Waves in Membrane Systems When Using Overlimiting Current Modes

**DOI:** 10.3390/membranes10080189

**Published:** 2020-08-16

**Authors:** Makhamet Urtenov, Natalia Chubyr, Vitaly Gudza

**Affiliations:** 1 Department of Applied Mathematics, Kuban State University, Krasnodar 350040, Russia; flash.wetal@mail.ru; 2 Department of Applied Mathematics, Kuban State Technological University, Krasnodar 350042, Russia; chubyr-natalja@mail.ru

**Keywords:** ion-exchange membrane, mathematical modelling, using overlimiting current modes, membrane systems, cation-exchange membrane, effect of the breakdown of the space charge

## Abstract

The study of ion transport in membrane systems in overlimiting current modes is an important problem of physical chemistry and has an important application value. The influence of the space charge on the transport of salt ions under overlimiting current modes was first studied in the work of Rubinstein and Shtilman and later in the works of many authors. The purpose of this research is to study, using the method of mathematical modeling, the reasons of formation and properties of the local maximum (minimum) space charge in membrane systems under overlimiting current conditions. It is shown that, in the diffusion layer of the cation-exchange membrane (CEM), the local maximum of the space charge appears due to the limited capacity (exchange capacity) of the membrane at a given potential jump, i.e., the local maximum of space charge appears due to the presence of a local minimum of space charge at the surface of the CEM. The local maximum of the space charge moves as a single soliton-like wave into the depth of the solution. Unlike real solitons, this charged wave changes its size and shape, albeit quite slowly. In the section of the desalination channel, the situation is completely different. First, the space charge of the anion-exchange membrane (AEM) has a negative value, so we should be talking about the local minimum (or the maximum of the absolute value of the charge). However, this is an insignificant clarification. Secondly, the space charge waves of different signs begin to interact, which leads to a new effect, namely the effect of the breakdown of the space charge. The dependence of the local maximum on the input parameters—the cation diffusion coefficient, the growth rate of the potential jump, and the initial and boundary concentrations—is studied.

## 1. Introduction

A thorough development of membrane devices has been done over the decades [1]. Of course, progress in separation through phase boundaries/interfaces has been accompanied by issues. The problem of ion transport across phase boundaries is one of the fundamental problems of physical chemistry and electrochemistry and is also important for membrane technologies. Studies [2,3,4,5,6,7] have shown the prospects of using intensive current modes.

The influence of space charge on the structure of the diffusion layer was first studied by Rubinstein and Shtilman [8]. Instead of the traditional equations of electroneutrality to the system of equations of the Nernst–Planck diffusion layer, the authors introduced the Poisson equation, and the ion-exchange membrane was taken as selective (the effective transport number of counterions was taken independent of current density). The problem was solved numerically. In the future, various mathematical methods and approaches to solving the Nernst–Planck–Poisson equations for electromembrane systems under extreme current conditions were developed [9,10,11,12,13,14].

The study of the non-stationary transfer of binary electrolyte in the diffusion layer is interesting because it allows us to determine the structure of the diffusion layer and its change over time, which is necessary, for example, for the asymptotic analysis of problems and the establishment of simple engineering calculation formulas for the dependence of the concentration distribution and electric field strength on the parameters of the problem. Research on non-stationary problems is limited to studies [15,16,17,18]. In these articles, the main attention is paid to the overlimiting potential dynamic mode and the analysis of the setting time depending on the parameters of the problem.

Studies [19,20] are devoted to the study of «shock electrodialysis», in which a deionization wave propagates through a microchannel or a porous medium with a sharp boundary between the concentrated and depleted zones. The deionization waves can be compared with the charge wave, since the deionization region actually coincides with the space charge region and a «powerful gradient» occurs in the desalination channel. Shock electrodialysis is a newly developed method for the desalination of water and deionization in micro-scale pores near an ion-selective element. In contrast to «shock electrodialysis», we study electro-membrane systems (the diffusion layer and the section of the desalination channel) with macroscale dimensions of the order of millimeters. In addition, it is shown that, in these systems, the interaction of charge waves is possible, up to their destruction (breakdown).

## 2. Materials and Methods

### Mathematical Model of One-Dimensional Non-Stationary Ion Transport in Membrane Systems 

Two electromembrane systems will be considered below: The depleted diffusion layer at the cation-exchange membrane (CEM), then x=0, the beginning of the diffusion layer, and x=H is the conditional boundary of the solution/CEM. Additionally, the section of the desalination channel, in this case x=0, is the conditional boundary of the anion-exchange membrane (AEM)/solution, and x=H has the same meaning. The transfer of 1:1 salt ions in both cases is described by the same equations—the difference in the boundary conditions at x=0. System of equations.

The non-stationary transport of salt ions for a 1:1 electrolyte is described by the following system of equations:

(1) $$\frac{\partial C_{i}}{\partial t}=-\frac{\partial j_{i}}{\partial x}\;i=1,2$$

(2) $$j_{i}=-\frac{F}{RT}z_{i}D_{i}C_{i}\frac{\partial \phi }{\partial x}-D_{i}\frac{\partial C_{i}}{\partial x}\;i=1,2$$

(3) $$\frac{\partial^{2}\phi }{\partial x^{2}}=-\frac{F}{\varepsilon_{a}}(C_{1}-C_{2})$$

(4) $$I_{c}=F(j_{1}-j_{2})$$

Here, (1) is the equation of material balance, (2) is the equation the Nernst–Planck for fluxes of sodium $i=1↔Na^{+}$ and chloride $i=2↔Cl^{-}$ ions, the charge number of cations $z_{1}=1$ and anions $z_{2}=-1$, (3) is a Poisson equation for the electric field potential, (4) is the equation of the current flow, which means that the current flowing through the diffusion layer is determined by the flow of ions, $\varepsilon_{a}$ is the dielectric permeability of the solution, F is the Faraday number, R is the universal gas constant, ϕ is the potential, $E=-\frac{\partial \phi }{\partial x}$ is the electric field intensity, and $C_{i}$, $j_{i}$, $D_{i}$ $I_{c}$ are concentration, flux, the diffusion coefficient of the i-th ion, and current density determined by the flux of ions, respectively.

Boundary conditions for the diffusion layer.

The boundary conditions for a diffusion layer consist of the following boundary and initial conditions:

(5) $$C_{1}\left(t,0\right)=C_{0},\;C_{2}\left(t,0\right)=C_{0},\;\phi \left(t,0\right)=0$$

$$C_{1}\left(t,H\right)=C_{1m}(t),\;\left(\frac{\partial C_{2}}{\partial x}+\frac{F}{RT}C_{2}E\right)(t,H)=0,\phi (t,H)=\Delta_{r}(t),$$

$$C_{10}\left(x\right)=C_{0},\;C_{20}\left(x\right)=C_{0},\;\phi_{0}\left(x\right)=0,$$

where $\Delta_{r}(t)$ is the potential jump. As a rule, $\Delta_{r}(t)$ is either constant, or $\Delta_{r}(t)=-d\cdot t$, where d is the growth rate of the potential jump and has the dimension V/s.

Boundary conditions for the desalination channel.

To model the ion transport, replace the boundary condition (5) with the following:

(6) $$C_{2}\left(t,0\right)=C_{2m},\;\left(\frac{\partial C_{1}}{\partial x}-\frac{F}{RT}C_{1}E\right)(t,H)=0,\;\phi \left(t,0\right)=0$$

## 3. Results

### 3.1. Reasons for the Formation of a Local Maximum (Minimum) Space Charge in the Extended Space Charge Region (Extended SCR)

**Figure 1:**
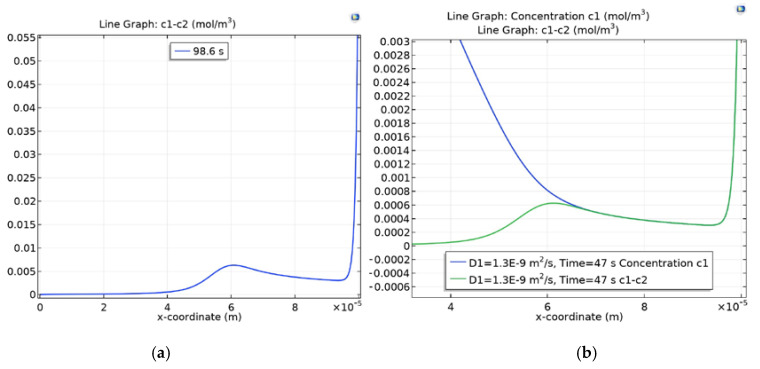
(**a**) Graph of the space charge in the diffusion layer; (**b**) Comparison with the graph of the concentration of $C_{1}$ cations near the cation-exchange membrane (CEM) when using overlimiting current modes in the dimensional form.

When the current passes through the membrane, the concentration of cations decreases and reaches its minimum at the left border of the border layer. When using overlimiting current densities, this minimum is preserved, but the minimum value is greatly reduced, and there are practically no anions in the area of the minimum point. The size of the space charge $\rho =F(C_{1}-C_{2})$ is almost completely determined by the concentration of cations $\rho \approx FC_{1}$(Figure 1b). Therefore, the space charge also has a local minimum other than zero at this point. The minimum value depends on the applied potential jump (or on the current that is passed through the system). On the other hand, in the depth of the solution, where the condition of electroneutrality is met, the value of the space charge is almost zero. Therefore, the value of the space charge must have a local maximum between them (Figure 1a). In [9,12], it is shown that $x^{*}=\frac{I_{np}}{I}H$; therefore, $\left[0,\frac{I_{np}}{I}H\right)$ is the region of electroneutrality, and $\left(\frac{I_{np}}{I}H,H\right)$ is the extended space charge region (SCR). In addition, in the field of electroneutrality, a balance is observed between the processes of diffusion and electromigration, and the currents of diffusion and electromigration (ohmic) are equal. At the same time, in the SCR, the electromigration is an order of magnitude greater than the diffusion one. Thus, the current pattern changes at the local maximum point.

Using the above assumptions, you can analytically determine the solution in the extended SCR. Indeed, we put in the system of equations $C_{2}(t,x)=0$, $j_{2}(t,x)=0$, $\left|\frac{F}{RT_{0}}z_{i}C_{i}\frac{\partial \phi }{\partial x}\right|>>\left|\frac{\partial C_{i}}{\partial x}\right|$, then we get:

(7) $$\frac{\partial j_{1}}{\partial x}=0$$

(8) $$j_{1}=-\frac{F}{RT}D_{1}C_{1}\frac{\partial \phi }{\partial x}$$

(9) $$\frac{\partial^{2}\phi }{\partial x^{2}}=-\frac{F}{\varepsilon_{a}}C_{1}$$

$$I_{c}=Fj_{1}$$

From Equation (7), we get:

$$j_{1}=j_{1}\left(t\right)=\frac{I_{c}(t)}{F}$$

Multiply Equation (9) by $\frac{\partial \phi }{\partial x}$, then, taking into account equation (8), we have:

$$\frac{\partial \phi }{\partial x}\frac{\partial^{2}\phi }{\partial x^{2}}=\frac{RT}{\varepsilon_{a}D_{1}}j_{1}$$

$$\frac{1}{2}{\left[\frac{\partial \phi }{\partial x}\right]}^{2}=\frac{RT}{\varepsilon_{a}D_{1}}j_{1}x+\beta$$

or

(10) $$\frac{\partial \phi }{\partial x}=-\sqrt{2\frac{RT}{\varepsilon_{a}D_{1}}j_{1}x+\beta }$$

where β>0 is the integration constant. From (8) and (10), we have:

$$C_{1}(t,x)=\frac{j_{1}RT}{FD_{1}\sqrt{2\frac{RT}{\varepsilon_{a}D_{1}}j_{1}x+\beta }}$$

or

$$C_{1}(t,x)=\frac{RT}{F^{2}D_{1}\sqrt{\frac{2RT}{\varepsilon_{a}FD_{1}}I_{c}(t)x+\beta }}I_{c}(t)$$

From this formula, it can be seen that $C_{1}(t,x)$ monotonously decreases in x in the extended SCR and reaches a minimum on its right border. In addition, as the current density increases, the minimum value decreases, which coincides with the numerical solution.

In order to understand the role and significance of the occurrence and growth of a local maximum, it is necessary to study its dependence on the input parameters of the problem, such as the initial and boundary concentrations, the rate of growth of the potential jump, the composition of the electrolyte solution, etc.

### 3.2. Dependence of the Local Maximum in the Diffusion Layer on the Boundary Concentration of $C_{1m}$

Consider the dependence of the local maximum on the parameter $C_{1m}$, which characterizes the exchange capacity of the membrane.

**Figure 2:**
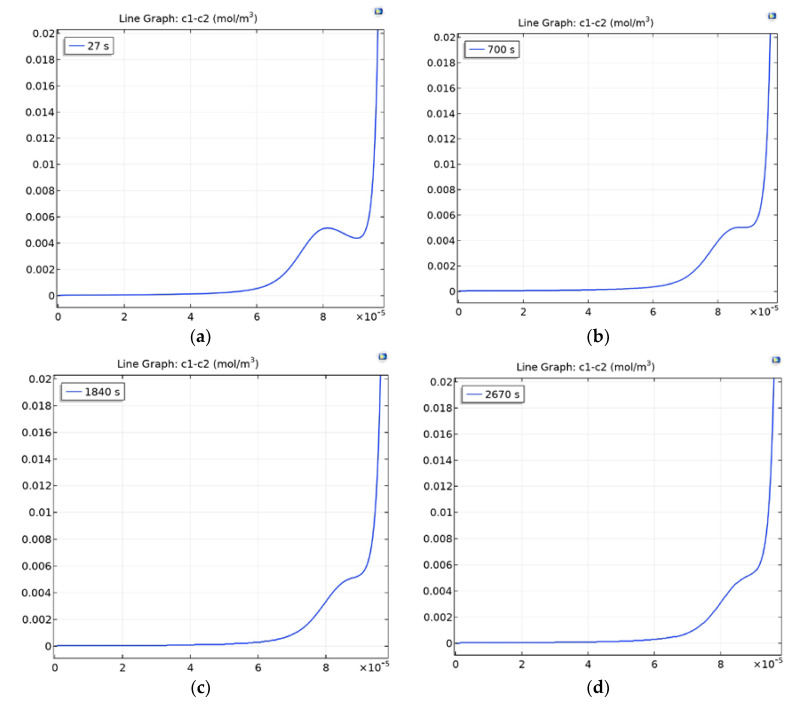
Dependence of the local maximum on the exchange capacity of the CEM (NaCl, $\Delta_{r}\phi =0.5$): (**a**) $C_{1m}=14$, (**b**) $C_{1m}=350$, (**c**) $C_{1m}=920$, (**d**) $C_{1m}=1335$.

The value in Figure 2 increases by the formula $C_{1m}=0.5\cdot t$, with the local maximum appearing and disappearing. The point of the local maximum is shifted to the right, but the value does not change much (Figure 2a,b). At the same time, the local minimum is gradually filled with increasing $C_{1m}$ (the value of the local minimum increases) and it is first compared and then surpassed (Figure 2c,d).

Thus, it can be concluded that the local maximum of the space charge appears due to the limited exchange capacity of the membrane at a given potential jump, i.e., the local maximum of the space charge appears due to the presence of a local minimum of the space charge at the surface of the CEM.

### 3.3. Soliton-like Charge Wave. Dependence of the Local Maximum (Minimum) Charge on the Growth Rate of the Potential Jump d

Consider a non-stationary model of transport in the diffusion layer of the CEM (1–5) with a linear growth of the potential jump $\Delta_{r}\phi (t,H)=-d\cdot t$.

The calculations are at times greater than a certain critical $t_{k}$, at which point the potential jump $\Delta_{r}\phi \left(t_{k},H\right)=-d\cdot t_{k}$ corresponds to the limiting current density and a local maximum of the space charge appears as a single soliton-like wave begins to move into the depth of the solution. Unlike true solitons, this wave has a charge. In addition, it changes its size and shape.

With a decrease in the growth rate of the potential jump d, the space charge wave becomes less pronounced, but remains.

### 3.4. Dependence of Charge Waves on the Cation Diffusion Coefficient

#### 3.4.1. Diffusion Layer

**Figure 3:**
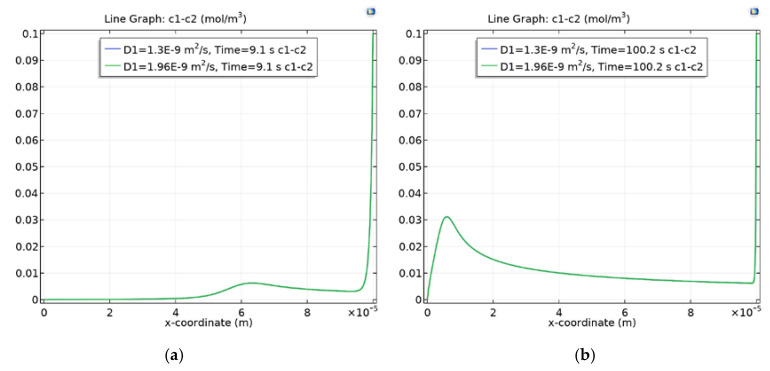
Wave charge when d=0.1 when t≈9c (**a**) and t≈100c (**b**). The blue and green lines (they merge with great accuracy) correspond to solutions of NaCl and KCl.

In order to study the dependence of the local maximum on the diffusion coefficient of the cation, solutions of NaCl and KCl are considered. Calculations show that, despite the difference in the diffusion coefficients of sodium and potassium by 1.5 times, the positions of the local minimum and maximum and their value in the diffusion layer of CEM differ slightly (Figure 3).

#### 3.4.2. Section of the Desalination Channel

Let us analyze the issue (1–4), (6). In the section of the desalination channel, the situation is completely different. First, the space charge of AEM has a negative value, so we must talk about the local minimum (or the local maximum of the absolute value of the charge). However, this is an insignificant clarification. Secondly, the space charge waves of different signs begin to interact, which leads to a new effect, namely the effect of the breakdown of the space charge.

**Figure 4:**
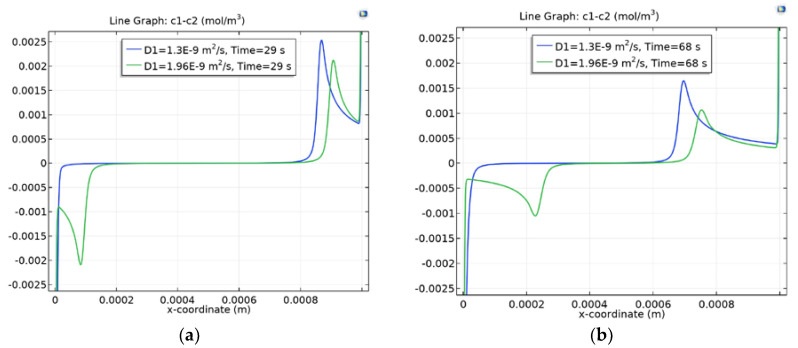

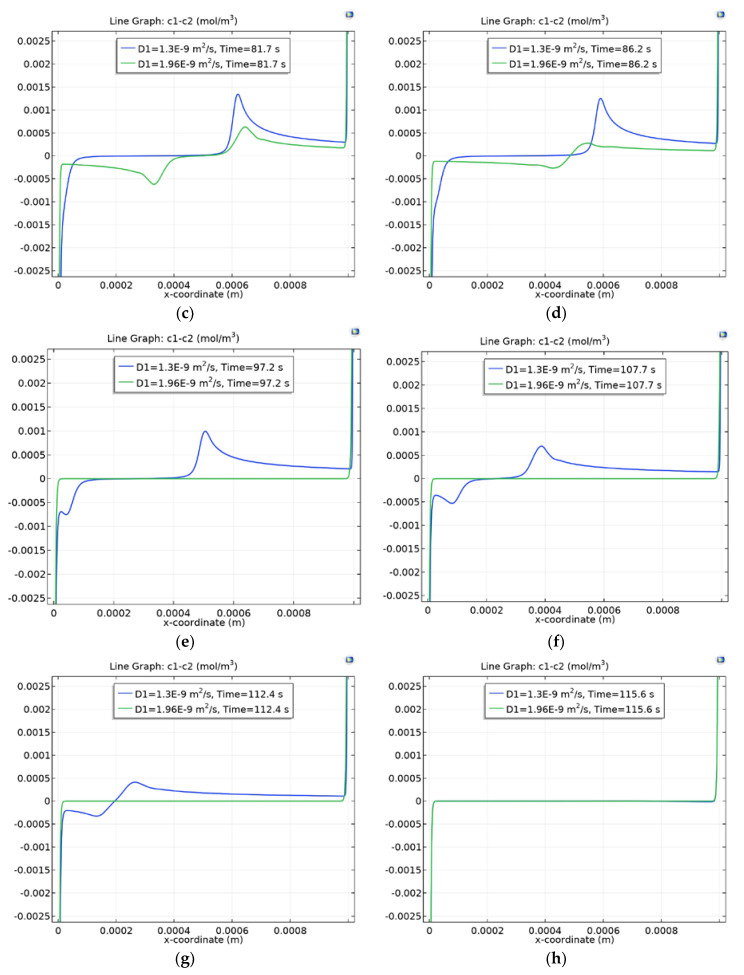
Graphs of the space charge at d=0.1, $C_{0}=0.1\;mol/m^{3}$. t≈29c (**a**); t≈68c (**b**); t≈82c (**c**); t≈86c (**d**); t≈97c (**e**); t≈108c (**f**); t≈112c (**g**); t≈116c (**h**).

Let us first consider the behavior of the local maximum in the KCl solution at d = 0.1. As can be seen from Figure 4a–f, two symmetrical soliton-like lone waves are formed, which move towards each other. Unlike real solitons, these waves have, as noted above, charges, the left wave negative charges and the right positive charges. At first, they practically do not interact, but as they approach each other they begin to attract and their speed of convergence increases and at the moment before contact there is a practical instantaneous breakdown, and they are discharged. A further increase in the potential jump does not lead to the formation of a new wave of local maximum, since the concentration of the solution practically becomes zero (Figure 4f–h), with the exception of narrow border layers in AEM and CEM, due to the fact that the concentration of anions and cations is maintained constant at the boundaries of AEM/solution and solution/CEM. As the potential jump increases, the width of the border layers decreases very slowly. A similar scenario is realized for the NaCl solution, except that the local minimum of negative space charge is generated much later (Figure 4f) than for the KCl solution. This is due to the fact that the current density in the first case is greater than in the second.

At d=0.005 in NaCl, a single wave occurs in the CEM, which reaches the region of negative space charge in the AEM, where there is no local minimum and maximum, and is discharged at 436 s. In KCl, two almost symmetrical waves are formed, one in the CEM and the other in the AEM, which meet almost in the middle and discharge at 282 c. Thus, the behavior of charge waves at d=0.005 in NaCl and KCl solutions is completely different.

### 3.5. Dependence of Charge Waves on the Initial Concentration of $C_{0}$

**Figure 5:**
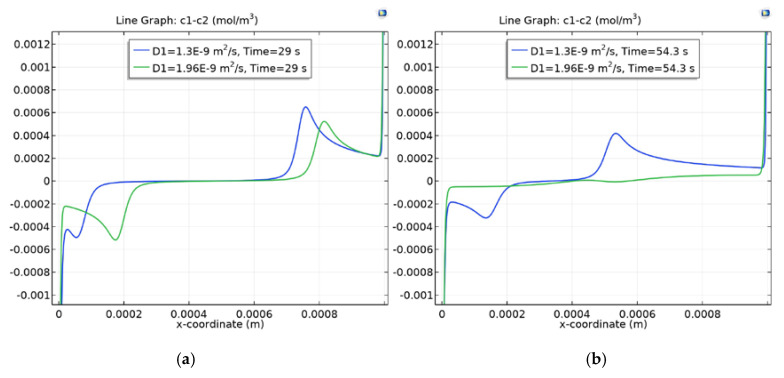
Space charge graph at $C_{0}=0.01\;\mathrm{mol}/m^{3}$. t≈29c (**a**); t≈54c (**b**).

The comparison of space charge graphs for $C_{0}=0.01\;\mathrm{mol}/m^{3}$ and for $C_{0}=0.1\;\mathrm{mol}/m^{3}$ (see Figure 4a and Figure 5a) at t=29 s shows that the less concentrated solution is desalinated faster and the charge waves are already very different, and, at t=54.3 s (Figure 5b), the charge waves for KCl began to discharge.

## 4. Conclusions

In this paper, the reasons for the formation and properties of the local maximum (minimum) space charge in membrane systems when using overlimiting current conditions are investigated. The depleted diffusion layer at the cation-exchange membrane and the section of the desalination channel are considered as membrane systems. It is shown that the local maximum and minimum of space charge appear due to the limited capacity of ion-exchange membranes at a given potential jump. It is shown that the local maximum of the space charge in the diffusion layer moves as a single soliton-like wave into the depth of the solution, slowly changing its size and shape. In the section of the desalination channel, the space charge waves of different signs begin to interact, which leads to a new effect, namely the effect of discharge (breakdown) of the space charge. The fundamental laws of this phenomenon are studied.
